# Psychology of Kant
*Immanuel Kant's sämmtliche Werke, (Rosenkranz,) Leipzig.


**Published:** 1859-01-01

**Authors:** 


### Art. III.—PSYCHOLOGY OF KANT.*

BY PROFESSOR HOPPUS.

(*Continued from* vol. xi. p. 521.)

We now proceed, from the Categories, to further developments. Our philosopher next gives us what he terms the *deduction* of the categories, meaning their derivation from their transcendental (*à priori*) source in the understanding itself, which applies them to the objects of our senses, since these objects constitute the *matter*, the categories the *forms* of our knowledge. For knowledge is possible only on two conditions—*intuition*, by which the object is given merely as bare phenomenon, that is, something presented to sense—and *conception*, by which it is thought. Kant is too chary of examples. But here he says: "A savage sees a house in the distance, but he knows not its use; he has the same object before him as another man has who knows it as a place to dwell in; but, in form, there is a difference; to the savage there is only intuition (*Anschauung*), to the other intuition and conception (*Begriff*), at the same time."† Now, the diversity of representations which are given in intuition (which is sensuous) are simply a mode in which we are passively affected: but the act by which this diversity is brought to unity in any case, does not come from our senses; nor is it included in the *pure* form of intuition—either space or time. In all cases of the union of different elements, whether they are given in our sensuous or in our thinking nature, there is an act of the understanding which we term a synthesis. All combination implies both diversity and unity. Sensible things furnish us with intuitions or sensuous representations:‡ understanding collects and unites them by its syntheses, and thus gives rise to empirical conceptions. In all our knowledge, sense and understanding must concur to produce the result.

In thus collecting into one diverse representations, the understanding, which is the faculty of cognition (knowledge,) must have the aid of imagination, memory, and consciousness. Reproductive imagination is required, because the putting together, mentally, of a diversity of sensible representations is an affair which must take place by successive steps: imagination must be always ideally reproducing, at each step, the parts which are successively put together, or they would be lost to us, and the picture would be imperfect. But such a reproduction involves the memory of what has already presented itself to our

* Immanuel Kant's sämmtliche Werke, (Rosenkranz,) Leipzig.

† Logik: *Einleitung*, V.

‡ *Vorstellungen; presentations* would here be a better word, though not usual.

senses. Again, memory implies the conviction of consciousness that the successive representations which present themselves in us are always identical. Thus every synthesis which the understanding makes when it thinks the objects of sense, is effected by the joint agency of imagination, memory, and consciousness —faculties which Kant regards as subservient to it.

The whole discussion on the "transcendental deduction" of the categories, or the legitimacy of their origin in the spontaneous activity of the understanding, and of their application to phenomena, is sufficiently obscure, although this portion of the *Kritik* was almost entirely recast in the second edition. It is closely interwoven with Kant's theory of consciousness—no longer now, as before, regarded as attaching to sensuous intuition, but as developed in the acts of the understanding—the faculty of thought. His speculations on consciousness are involved in considerable difficulty; and the changes he made, in different editions, on this subject, indicate something like a want of reliance on his own statements.

Here we must remind the reader that we have already glanced at our author's doctrine of *internal sense*—that is, of the intuition of self and our internal state, of which the *form* is time, since all that passes within us must take place in time. We have seen that he has identified this internal sense of self and its states, with consciousness (*Bewusstseyn*); and he says that, by means of it, the phenomenal reality of myself and of my internal state are clear to me. In the Transcendental Æsthetic, he described the consciousness of oneself, or "apperception," as the simple representation of the *ego*;* and he adds, that the subject (mind) intuits or views itself according to the manner in which it is internally affected, or as it appears, not as it is—that is, the mind is presented to itself phenomenally only, like any phenomenon of external sense. Hence this sort of consciousness is an affair of sense, and not of understanding; we are as passive in it as we are when a sound salutes our ears, or the sun dazzles our eyes.

In his doctrine of the understanding, however, Kant presents to us a very different view of consciousness. It is now an affair of thought, not of sense. The terms, indeed, in which these two aspects of the subject are expressed (*Bewusstseyn—Apperception*) are used in reference to both, which tends to perplexity rather than to clearness;† but when he wishes to *distinguish* the con-

* Das Bewusstseyn seiner selbst (Apperception) ist die einfache Vorstellung des Ich, etc., *Vid. Kritik* (Rosenkranz), *Supplem. XI.* This whole passage contains the main development of Kant's theory of consciousness as identified with the internal sense.

† Perhaps some light may be thrown on this obscure portion of the *Kritik*, by a reference to the two different views which have been entertained respecting consciousness of thought from the consciousness of sense, he names the former *apperception,* the latter *internal sense.* We give a very few brief quotations:

"The *I think* (Ich denke) must accompany all my representations, or else they would be in relation to me nothing. Representation given previously to all thought is called intuition. All the diversity of intuition has a necessary relation to the *I think*, in the subject (mind) in which this diversity is found. But this representation, *I think*, is an act of spontaneity; that is, it cannot be regarded as belonging to mere sensibility. I call it pure apperception. It is a self-consciousness to which all my representations belong, and in which I unite them.* I am conscious of a necessary *à priori* synthesis of my representations, which is called the original synthethic unity of apperception. In systems of psychology, the internal sense is commonly held to be one with this faculty of apperception; while we, on the contrary, carefully distinguish them."

"How the *ego* which thinks is distinct from the *ego* which intuits (views) itself, and is yet one and the same with it, as the same subject—how I as *thinking* intelligence know myself as an object *thought*, in intuition, like other phenomena—not as I am in myself, and as considered by the understanding, but merely as I appear?—is a question neither more nor less difficult than the question, How can I be an object to myself?—how can I be an object of my intuition and inward perceptions? Such, however, is the fact."

"As regards internal intuition, we know our own subject (mind) as phenomenon, and not as it is in itself. On the other hand, in the transcendental synthesis of manifold representations, consequently in the synthetical unity of apperception, I am conscious to myself, not as I appear to myself, nor as I am in myself, but only *that I am.* This representation is a thought, not an intuition."

"I do not see why so much difficulty should be found in admitting that our internal sense is affected by ourselves; every act of attention exemplifies it. . . . . . The *I think* expresses the act of determining my own existence; but the *mode* in which it is determined is not given in this spontaneous act; my existence remains determinable in a purely sensuous manner, as the existence of any other phenomenon." †

sciousness among English writers. Some hold that consciousness is that power of inward self-survey, by which we make the conception of *self* and our internal states an object of attention and thought. Others say, that the having any sensation or thought, or being in any state whatever, however passively, is consciousness; and these would not allow that the term should be restricted to the peculiar state in which we are when we are making the idea of self and our internal modifications a special object of attention. Perhaps the two views admit of being somewhat harmonized, but our sphere forbids our entering on this subject. Possibly Kant intended both a general and a special consciousness: he evidently saw no contradiction in his theory, as given under the head of sense, and as given here under the head of understanding, though he was quite alive to the mystery of the fact that the mind *can* and *does* direct itself to an observation of its acts and states, as its own.

* Wolf says (*Metaphys,* cap. iii. § 194,) "Die Verbindung der Vorstellung mit dem Bewusstseyn heisst *denken.*"

† Kritik der r. Vernunft: *Deduction der reinen Verstandes-begriffe. Ausg.* 2.

It is evident from these statements and others that might be quoted, that Kant held with *two* principles in consciousness, which were always found co-existing. This union of both has perhaps saved him from wholly contradicting himself on this subject.* The internal sense, the instrument of psychological observation, is not the same thing as the faculty of pure apperception. It is in the same relation to this latter as external sense is. The *ego* as a thinking subject is to be distinguished from the *ego* as an *object* of intuition and thought. Self-consciousness, or mere intuition, is not yet knowledge of self: this knowledge is the product of the operation of the understanding on the data of internal sense. Kant strenuously maintains that "the '*I think*' is, in all acts of consciousness, one and the same;" and that, unaccompanied by it, no representation whatever, and therefore not the simple representation of the *ego*, "can exist for me." He inquired into the contents of consciousness, and aimed to show what there is in it that is sensuous and empirical, as belonging merely to the internal sense, and what there is *à priori* and belonging to the understanding. The internal sense contains no synthesis; therefore the diversity of representations or intuitions which present themselves in it would have no meaning, unless to this diversity there were superadded a certain unity. The synthetic process which reduces to unity the various matter furnished by intuitions, both pure and empirical, is a spontaneous operation of the understanding; and this unity has its principle in the unity of consciousness—that is, in the pure apperception "I think." The diversity of representations to the mind produce only "empirical apperceptions:" the unity comes from the synthetic power of pure or primitive apperception, or the self-consciousness which attends all thought, and is its proper seat. Kant sometimes terms this principle of unity in which all representations belong to one identical *ego*, the transcendental unity of apperception, as being the principle of all knowledge, and therefore of the categories—it is indeed the highest principle of the understanding itself. It is primitive and absolute, the basis of all the activity of the understanding, a condition of all knowledge and of all intuition.

But from this subjective unity of consciousness by means of which everything is referred to the *ego*, we are to distinguish that *objective* unity by means of which the variety of intuition is referred to an object. The act of understanding by which the latter unity is effected is the judgment. Objective knowledge is therefore subjected to the primitive elements of judgment—that is, to the categories. These, though only simple forms of thought, have an objective validity in their application to phenomena, which

* M. Cousin, however, maintains that the two views are in absolute contradiction, and wonders that no critic of Kant has ever pointed this out. *Leçons sur Kant*, 5.

these forms determine. Our experience of all nature becomes possible only by means of these categories, for that experience must be in accordance with them. As the *à priori* conditions of experience, they are not derived from nature—they constitute its laws. The phenomena of nature have reality, therefore, only in relation to our sensuous faculties: their laws only exist for our faculty of thought. We know nothing of things in themselves or their laws. The laws of phenomena are all that we know, and these are the laws of thought. Nature is nothing but the systematic aggregate of phenomena, such as we conceive them in virtue of the laws of our faculties of sense and understanding.

2. The Analytic of Principles (*Analytik der Grundsätze*) next follows. It is to be a guide to the faculty of judgment in applying to phenomena the categories, which are the necessary conditions of *à priori* principles, as will appear; for understanding includes both conception and judgment; which latter faculty brings particular cases under some general rule or form.

The *schematism* of the pure conceptions of the understanding (categories) is the first point to be noticed under this "transcendental doctrine of judgment." When an object is brought under a conception, the representation must be homogeneous with the conception. The empirical conception we form of a *plate* is homogeneous with the pure geometrical conception of a circle; for the roundness which is *thought* in the latter, is *intuited* (seen) in the former. But the pure conceptions of the understanding are not at all homogeneous with sensuous intuitions, and can never be discovered in them. How then can phenomena be brought under the categories? Take, as an instance, the second of the categories of relation,* which is *causality*. This is not an object of sense. We do not see causes: we only think them. We simply see changes and successions of phenomena—that is, we see objects in different circumstances. How then are we entitled to unite the pure conceptions of the understanding with these intuitions of sense? There must be, says Kant, some third thing, some medium, homogeneous with the special category and with the particular object which is to be subsumed under it. This medium must be both sensuous and intellectual.

This medium is *time*. It is the "transcendental schema;"† for it is the form or condition under which all the objects of sense must present themselves to us. They all appear in time; and all conception and thought must also go on in time. Time is the common form both of external and internal sense. It is therefore the uniting bond of both; and its transcendental determi-

* See the Table of the Categories in our Number for July, 1858.

† Aristotle uses this term (σχημα) for the figures of the syllogism.—*Analyt. Prior.* Lib. i. cap. 6.

nations, as we shall presently see, render possible the *schematism* which enables us to fit on, as it were, the categories to the corresponding phenomena. Many of our readers perhaps would see little difficulty in saying of a certain object that it was a unity (one,) or of another that it was a totality (whole) of parts, and the like. "Common sense" would be sufficient here, as usual. Kant, however, speaks almost with awe of this schematism as a sort of mystery, "an act hidden in the depths of the human soul." It is, at all events, an ingenious subtilty, founded on a true view of time as the background of all sense and thought, whatever may be its merits in detail. Of course it is, as usual, loaded with technicalities.

A schema is not an image. Five dots form an image of the number five. But we can only *think* number in general: we cannot make an image of anything that is not special. Number in general is the *schema* of the conception of any particular number of which an image may be formed. So also no image can be made of a triangle in general. It is a schema of the understanding, which contains under it all the species of this figure, of the several conceptions of which images may be formed. Such images come from the empirical power of productive imagination. The schemata of sensuous conceptions, as figures in space (triangle), arise from the pure imagination *à priori*, which renders the images possible; but the schema of a pure conception of the understanding (category) is, of course, not the schema of an image: it is simply the pure synthesis expressed in the category conformably to a general rule. It is a transcendental product of imagination determining our internal sense according to the conditions of time, in respect to phenomena, in harmony with the unity of consciousness. The transcendental schemata of the categories, and the sub-schemata of the sub-categories are as follows:

The schema of *Quantity*, as a pure conception of the understanding, is *number*, which comprises the successive addition of unities of the same kind in a *series of time;* so that number is the synthesis of all the variety of our homogeneous intuition in general. This view applies equally to unity, plurality, and totality: for unity as applied to an object means that it is taken one time, plurality more than one time, and totality a unity of times.

The schema of *Quality* marks the content of time, or *existence in time*. Reality as a pure conception corresponds to our sensations and the phenomena which occasion them. Every sensation has a degree by which it fills time, more or less, until it vanishes into negation. We may see a brilliant light: here is something in time, or which fills time, a reality. The sensation may diminish, the light becoming fainter and fainter: here is limitation, or a transition from time filled to time empty; and when the light has vanished we have a negation of the reality, or non-being in time.

The schema of Relation has reference to order or *arrangement in time*. Subsistence has for its schema permanence in time, or reality considered as the substratum which remains while all else changes. Time passes not, says Kant, it does not change. Hence what is unchangeable in objects corresponds to time; and permanence in time is the most general conception of substance, which is the unchangeable in existence. Causality, again, points to priority and succession in time, so far as this is subjected to a certain rule. Community or reciprocity, which is the mutual causality of substances, or the interagency of correlates, has for its schema the co-existence or contemporaneousness of their reciprocal determinations, according to a rule.

The general schema of Modality is the *mode of connexion or non-connexion with time*. Possibility involves such an accordance with the general conditions of time, as that a thing is conceivable. A thing is always possible to us when it is conformable to the conditions of any one time; but two contradictory propositions exhibit what cannot belong to the same thing at the same time. Existence* is schematized by the contemplation of anything as being in a certain determined time. The schema of necessity is exemplified in the being of the object in all time.

As we have from the beginning added our own illustrations where it seemed desirable, we will here give the above theory in a Table, which Kant has not done. It will already have been noticed that the schematism closely follows the order of the categories.

SCHEMATISM OF THE CATEGORIES.

1. Quantity, (series in time.)
   Unity, (one time.)
   Plurality, (more than one.)
   Totality, (unity of times.)
2. Quality, (content or matter of time.)
   Reality, (time filled.)
   Negation, (empty time.)
   Limitation, (transition from former to latter.)
3. Relation, (arrangement in time.)
   Subsistence and inherence, (co-existence with time.)
   Cause and effect, (succession in time.)
   Reciprocity of action, (contemporaneousness of mutual influence.)

* The term here used is *Wirklichkeit*, reality. In the list of Categories, both in the Kritik and in the Prolegomena, *Daseyn* is the word for this second sub-category under Modality, and *Realität* for the first sub-category of Quality. Kant, in using this latter term, in his Schematism, makes it synonymous with *Sachheit*, matter of fact. It is evident, however, that he attached no importance to these verbal variations. Existence is a matter of fact, both in distinction from a mere negation, and a mere possibility.

4. Modality, (mode of connexion or non-connexion with time.)
   Possibility and impossibility, (conceivableness of being in some time or not.)
   Existence and non-existence, (being in a certain time or not.)
   Necessity and contingency, (inconceivableness of not being at all times, and the reverse.)

Such, then, is the "schematism" of the understanding. Agreeably to his twofold view of consciousness, Kant says it is "simply the unity of the varieties of intuition in the internal sense, amounting indirectly to the unity of apperception, as a function which corresponds to the internal sense as a receptivity." He adds that as the categories merely serve to subject phenomena to these forms of synthesis, they are only capable of empirical use, though they are themselves *à priori*, being derived from the mind itself, and not from experience. Thus the categories are limited by sense; for the schema, in any case, is properly the phenomenon, or the sensuous conception of an object in harmony with the category. Number is phenomenal quantity, sensation is phenomenal reality, and the like. The categories, without schemata, are mere empty functions of the understanding, they derive their significance as well as their restriction from sense.

2. In immediate connexion with the above schematism, arise what our philosopher terms *the system of all principles of the pure understanding.* Having shown the general conditions under which the categories are applicable to our sensuous experience, so as to enter into synthetic judgments (propositions), he next proposes to exhibit systematically these judgments—namely, those which the understanding produces *à priori*, ultimate principles not being derived from any higher or more general truths. These principles are nothing more than rules for the objective use of the categories.

As these principles are synthetical, and not analytical, Kant here adverts to the distinction with which he set out. The judgment (proposition) *body is extended* is analytical, the predicate being already contained in the subject. *Body is not extended,* would therefore be a logical contradiction—it would be saying that something extended is not extended. The "principle of contradiction," therefore, is the sufficient test of the truth of all analytical judgments. But of synthetic judgments this principle is not a test, it is only a *sine quâ non. Every event has a cause,* is a synthetic judgment; for the conception "cause" (the predicate) is not contained in the conception "event" (the subject). It is also *à priori,* for it is not an induction from experience, but is a universal and necessary truth. No doubt its predicate does not contradict its subject; so far well, but this says nothing to its validity. Kant says, again, that there must be a medium in which alone the synthesis of subject and predicate can unite—it is that in which all our representations are contained—namely, the internal sense, and its form *à priori, time.* The synthesis depends on the imagination; their synthetical unity in the proposition (judgment) on the unity of consciousness. This appears to mean that such truths—those which can neither be proved nor denied—have their seat and their self-evidence in the inmost convictions of our consciousness. We believe such propositions just because we cannot help believing them.

The *principles* which we have now to detail relate, severally, under the categories, to the following heads, which are given in Kant's usual peculiar and technical style:

| | |
|---|---|
| 1. QUANTITY.<br>Axioms of Intuition. | 2. QUALITY.<br>Anticipations of Perception. |
| 3. RELATION.<br>Analogies of Experience. | 4. MODALITY.<br>Postulates of Empirical Thought. |

1. *Axioms of Intuition*—that is, the laws or ways in which objects can be presented to us, have for their principle, that *all phenomena are extensive quantities;* they have magnitude in space, or are conceived of as having continuity in time: for space and time are the conditions *à priori* of all intuition. All phenomena are in them, and can only be received into our empirical consciousness (that is, we can only have an actual experience of the things around us) by means of a successive synthesis, whereby is generated the representation to us of a determined space or time, more or less, the parts rendering possible and preceding the whole. A line, for example, however short, must be drawn in thought, part after part, if it is to be represented to our mind. So also of time, short or long—we think of its successive progression. On this successive synthesis depends the generation of geometrical figures. Geometry furnishes examples of certain axioms of intuition; as *between two points only one straight line is possible,* and *two straight lines cannot inclose a space.* Here, of course, we have to do with extensive quantities—that is, magnitudes. It is evident that the sub-categories of quantity, which are unity, plurality, and totality, have always to do with phenomena as presented in space, or numbered in time—that is, they all express magnitudes conceived of as measurable, and therefore to be called "extensive."

2. *Anticipations* of Perception.*—By this expression Kant means our previous knowledge of a certain general characteristic of our sensations. He does not here allude to the universal *à priori*

* Kant says the προλῆψις of Epicurus suggested to him this term.

conditions of space and time as necessary to our perception of phenomena. What to us appears real in these phenomena corresponds to the sensations they produce in us. Hence the principle of the *à priori* knowledge or anticipation referred to is, that *all phenomena, as represented by our sensations, must have intensive quantity, or must present themselves as of a certain degree of reality.* Perception he defines as "empirical consciousness, or a consciousness containing an element of sensation." We refer our own sensuous affections to some external object; and in so doing we ascribe to it degree, or *intensive* quantity, a degree of effect on sense. Kant uses the term "intensive quantity" for a quantity which is "apprehended as a unity only, or in which plurality can be represented only by gradual approximation to negation = 0." For our perception of external things takes place through sensation, which has no extensive quantity. There is in it no successive synthesis from part to part till we reach the whole as in measurable magnitudes. Our sensations are wholly subjective; they are our own feelings, though they represent qualities of body. Their only quantity is the degree of their intensity, and hence we call them intensive magnitudes. They may vary from any degree, by diminution, till they vanish. A sensation has no parts, and exists only the moment we feel it. If it diminishes or increases, it becomes, as it were, so many varieties of sensation, perhaps of inappreciable succession—but still, in fact, so many intermediate sensations between the highest degree and zero. There is not here one continuous quantity, as in extensive magnitude, but only a concatenation of many possible degrees ascending or descending, each of which is less different from another than the sensation in its original degree is from zero. A rainbow affects our eyes, say, in its most vivid state; it becomes fainter and fainter till it vanishes. A body feels hot to our touch, it gradually feels less hot, and at last we have no sensation of heat from it. Though our sensations do not admit of linear admeasurement, like extensive magnitudes, yet numbers may be used to determine *à priori* certain intensive phenomena. Thus, the light of the sun must be anticipated to produce a sensation—say "200,000 times" stronger than that of the moon. Heat, again, is measured by the thermometer. On account of quantity and quality being thus determinable, Kant regards the synthesis belonging to these categories as mathematical or *constitutive.* He terms the third and fourth principles, to which we now proceed, *regulative,* as they only concern, as we shall see, the relations of existing phenomena—not the very phenomena themselves in their magnitudes or degrees.

3. The general principle of the *Analogies of Experience* is that *experience is only possible through a necessary connexion of our*

*perceptions;* in other words, we must have in our minds a fixed synthesis of phenomena, in order to have an experimental knowledge of them. Experience is a synthesis of our perceptions, which is not contained in our perceptions themselves. I see the woodman's axe fall: I afterwards hear the sound of the blow. Here are two perceptions, one from sight, one from hearing. My experience teaches me to connect them as cause and effect; but the perceptions themselves do not contain this synthesis or conjunction as involving a necessary bond between the phenomena. In Kantian phrase, we have here a synthesis which contains the "unity of the diversity of these perceptions, in our consciousness." The two events only present themselves as empirically related in time, but with no sense of necessity. Experience, however, is only possible by means of the necessary connexion which the mind itself gives to its perceptions. Kant expresses the general principle also thus:—All phenomena stand, *à priori*, under rules of the determination of their relationship to each other in time.

The three modes of time, says our author, are *permanence, succession,* and *co-existence;* corresponding, in the categories of *Relation,* to substance and accident, cause and effect, and reciprocity between agent and patient. Hence the three modes give the three analogies of experience—that is, the three laws for determining all the relations of phenomena in time, laws prior to experience and rendering it possible.

(1) The first analogy of experience is the principle of the *permanence of substance.* In all the changes of phenomena, the substance or object changes not: its quantity remains the same; the changeable is only the mode in which the permanent exists. The substratum or substance corresponds with time in general, which changes not; the accidents alone change in time. Substances are the basis of all the time-determinations which phenomena present to us. Accordingly, the permanence of substance is a necessary condition under which alone phenomena as things or objects are determinable in a possible experience.

(2) The second analogy of experience is the principle of the *succession of time, according to the law of causality;* that is, all changes take place according to this law of the conjunction of cause and effect. One phenomenon as effect succeeds another as cause—thunder succeeds lightning. We unite the two perceptions, not by the senses, but by the synthetic faculty of imagination, which determines the relation of the phenomena in our internal sense in regard to time. What is further required is, that the order of the phenomena should be determined, that is, which must precede, and which follow. This relationship between them is a pure conception of the understanding by which cause determines effect as consequence. It follows that experience is only possible in so far as we submit phenomena to this law of causality. True it is that, in our apprehension of any phenomenon, the parts always follow each other, though they do not so succeed each other in the object. I see a house: in this apprehension, I may begin anywhere, at the roof or at the foundation. In this empirical intuition, I may apprehend the manifold by going from left to right, or the reverse. But the parts do not *so* follow each other in the object; there is no determinate objective order in this case; nor is any particular order thought as necessary in this synthesis of mere apprehension. Not so where an event B succeeds its antecedent A, as when the explosion of the cannon succeeds the flash. I here derive the subjective order of my apprehension from the objective succession of the phenomena; and thus my apprehension proceeds according to a rule. It is, therefore, justly supposed that every event is preceded by something which causes it, that is which it follows according to a rule; and in this condition experience is possible. This principle of causality is not a mere induction; if so, it would be wanting in universality and necessity. Why do we find this conception of cause in our actual experience, but because it is *à priori* in the understanding? No doubt it was first elicited on occasion of our empirical perceptions; but this conception once gained, we yield to it as the *à priori* condition of the synthetic unity of certain phenomena in time, and as the condition of all our experience of actual causes and effects. The principle of the succession of effect to cause still holds, even where the effects are apparently simultaneous with the cause. The relation remains, even where no time perceptible to us has elapsed; for had not A existed, B would not have arisen.

(3) The third analogy of experience is the *principle of co-existence according to the laws of reciprocal agency.* All substances, so far as they can be perceived in space as co-existing, are in mutual connexion with each other. Our mind views the changes of state which substances undergo, as having their causes in other substances; so that nothing exists in a state of isolation, but there is a mutual intercommunion among objects, and the active forces which render matter what it is are always in a reciprocal action among themselves. Things co-exist when the perceptions we have of them can be taken in any order; when, for instance, we can go from A through B, C, D, to E, or retrograde from E to A, or proceed in any other way. Now this, as we have seen, we cannot do in the case of cause and effect, which necessitates a certain succession: we can only place A before B, we cannot reverse the order in causation. But on the other hand, I can look first at the earth, then at the moon, then at a star, or I can look at them in any order as I please. Hence the earth, the moon, the star, are contemporaneous. I am entitled to say so, simply because my perceptions of these objects can thus follow each other reciprocally, in any order. The synthesis of the imagination would only go from one of these objects to another, but would not show their co-existence. A conception of the understanding, or the category of reciprocal sequence, is necessary to justify us in representing co-existence as phenomenally objective. Hence the co-existence of substances in space can only be cognised under the pre-condition of their reciprocal action; and this is, therefore, the condition of our experience of things. It is only by observing that the order of our synthesis of the objects is arbitrary and indifferent, that we can say they co-exist in one and the same time. Kant says it is evident, in our experience, that it is only the continuous influences in all parts of space that can lead our senses from object to object: the light which plays between our eyes and the heavenly bodies effects a mediating reciprocity between them and us, which shows their co-existence with ourselves. It is the relation of reciprocal causality, or of a certain interagency, which alone renders possible our experience of the simultaneous existence of things. Only thus—by this community, can objects produce in us a system of corresponding perceptions, and a real whole.

Such are the three "dynamical" relations which involve all others. Objects must be related to each other as *inherent* one in the other, or as *consequential* to each other, or as *combined* into a whole; and the three analogies of experience determine things according to the three modes of time—*duration, succession,* and *simultaneousness.*

4. The *Postulates of Empirical Thinking.* The general principle here is, that everything which can be known in our experience, must be known under a certain modification of the connexion between the subject and the predicate of the proposition in which we state our knowledge of it. Either A may be B (possibly); or A is B (the fact exists); or A must be B (necessarily). The categories and judgments of modality do not add anything to our conception of things; they only show how that conception is related to our faculty of knowledge. The postulates of our empirical thought are nothing more than explanations of possibility, actual existence, and necessity, as applied to our experience of things.

(1) Of these postulates, the first relates to possibility; and here the principle is: *that alone which agrees with the formal conditions of experience (intuition and conception) is possible.* The meaning is that for any event or object to be thought as possible to occur or exist, it must not violate the laws either of sense or understanding. Logical possibility merely demands consistency in our conceptions: thus that a triangle should have four angles involves a logical contradiction. But Kant here refers to a possibility that is real, or in things. The conception of a figure made up of two straight lines implies a synthesis that is void; as it can refer to no object, not admitting of being constructed in space. It belongs not, therefore, to experience. So the conception of a body constantly present in a portion of space without occupying it, equally disagrees with the conditions and determinations of space. Now these latter apply to all possible things, because they contain *à priori* the form of our experience of objects in general. Such conceptions, therefore, as the above have no objective reality; that is, they cannot be realized in experience—they are opposed to its known conditions.

(2) The second postulate is that, *in order for anything to be real, or actually existing, it must cohere with the material conditions of experience* (*perception, and therefore sensation*). In other words, what has actually affected our senses is not merely possible but really exists. Perception by conscious sensation is here required, according to the analogies of experience. From the mere conception of a thing, however complete, and however accordant with the conditions of a possible experience, we can never conclude that it exists. If this conception of it precedes the perception, all we can say is, that it is possible; but when our perception of a thing presents *matter* to the conception of it, we have the true mark of its reality. Our personal knowledge of the existence of things, therefore, reaches as far as our perceptions, and what may be immediately inferred from them according to the laws of experience, extend. Sometimes we know the existence of a thing comparatively *à priori*, and mediately; that is, when it attaches itself to certain perceptions according to the analogies of experience; so that we can reason from a thing which we do perceive to the thing we do not perceive. Thus we know the existence of a magnetic matter in bodies, by the actual perception of the attracted iron filings, though we cannot perceive the matter itself.

(3) The third postulate is as follows: *that exists necessarily, the coherence of which with the real is determined according to universal conditions of experience.* The necessity here spoken of is not mere formal and logical necessity, as when one conception must imply another; it relates to necessity of existence, which can only be known in connexion with an object of perception; and the only existence thus conditioned is that of effects from given causes, as when A being given in perception, B follows in conformity with the laws of causality. Hence the mark of necessity is to be found only in that law of a possible experience, that everything which happens is determined *à priori* by its cause. We therefore only know the necessity of those effects in nature the causes of which are given to us; so that this necessity only regards the relations of things according to the dynamical law of causality. Nature could not exist, unless all that happens were subjected to a hypothetical necessity; that is, every effect must happen if the cause be present. Hence "nothing happens by blind chance," and "there is no such thing as fate," are *à priori* laws of nature, meaning that all is conditioned by causation. This principle forbids any gaps or breaks in the series of phenomena (*in mundo non datur hiatus*), and any leaps in nature (*non datur saltus*); for there must be a continuity in all causes and effects; and no void can be admitted as a part of empirical synthesis.

It may be added that while Kant, as we have seen, terms the axioms of intuition and the anticipations of perception mathematical or constitutive, because they relate to Quantity and Quality, which are extensive and intensive magnitudes—he calls the analogies of experience, and the postulates of empirical thought *dynamical* or regulative; because, since they concern the Relation and Modality of objects, they bear, not on the nature of objects, but on the principles which affect their existence.

Kant concludes the above systematic representation of "all the synthetical principles of the pure understanding," by reminding us that all which he has set forth shows that the categories, in themselves, are not cognitions (knowledge), but merely *forms* of thought for making knowledge from given intuitions (sensuous representations) of objects. Our external senses, alone, can exhibit the conceptions of understanding in objective reality. If we say "all contingent existence has a cause," this only tells us that, without this relationship to cause, we do not at all comprehend the existence of the contingent. We could never know the existence of such a thing through the understanding and *à priori*. The question is not, how may things exist in themselves, but how *must* they exist if we are to *know* them? The categories only furnish us with laws of the possibility of experience, and of our knowledge of objects as given in our empirical intuition. The final result of the whole is, that all principles of the pure understanding are nothing more than principles *à priori* of the possibility of experience; that all synthetical principles *à priori* relate and apply to experience alone; and that their very possibility rests entirely on this relationship.

Kant's systematic application of the twelve categories to "principles" here ends. As a kind of appendix to the second postulate, he adds some paragraphs by way of refuting Material Idealism, which pronounces the existence of external objects either doubtful or untrue. Descartes said there was nothing absolutely unquestionable but the one empirical assertion: *I am*. Berkeley denied the possibility of all material existence. Kant calls this "dogmatical idealism." His own theory—that we know phenomena only, though real things exist—he terms "critical idealism," as being the result of the criticism of reason. Yet, by his making space and time to be simply psychological, and not independent of our sensuous faculty, his realism is irreconcilable with the idealism which characterizes so strongly his whole system. Space and time, he says, are only in *us;* they do not belong at all to things themselves, we only place phenomena in them. They are only "receptivities" (capacities) of our faculty of sense. This vital inconsistency was not only seen by the Wolfian school; Fichte a disciple of Kant saw it, and started from this point to his own egoistic idealism. Whatever may be thought of Kant's arguments in favour of realism, against Berkeley or Descartes, they are out of all harmony with his idealism of space and time. He strangely says, however, that if space and time were not wholly and alone in *us*, the external world would be a nonentity! On the contrary, can anything be plainer than that if, as Kant admits is the case, things really exist externally to us, and are not, as Berkeley said, merely our ideas and feelings—then space and time must be something more than mere forms of our sensuous faculty, otherwise the very existence of objects in themselves must depend on our own existence. If the material world really exists, it must exist in space and time: if not, the material world is but an illusion.

Our philosopher further argues that Descartes' principle of the certainty of our own personal existence from consciousness, proves the existence of phenomena in space, which involve something permanent as the cause of our perceptions. Our internal experience is only possible under the previous admission of external experience. My internal experience (consciousness) only gives me representations, but these changeful representations which are presented to me imply of necessity something permanent. Now this permanent must be something without me, and not a mere representation. Nothing permanent corresponds to our notion of body except *matter*. Therefore my existence in time can only be determined (made known) to me by the existence of something out of me; and the material world exists. "The consciousness of my own existence is at the same time an immediate consciousness of the existence of things without me." It is now generally admitted, with Kant, that the agency of external objects is the means by which all our faculties are excited to action. This is obvious with regard to our senses; and if we had no senses, how could any of our other powers have, at the outset, materials to work on? Thought itself must be first elicited by sense. A human being without all sensation could not know even his own existence, under the present order of things. The *me* and the *non-me*, the subjective and the objective, are therefore inseparably correlated in all our knowledge. There is a question, however, whether the term "consciousness" (*Bewusstseyn*) ought to be indiscriminately applied to our knowledge of ourselves and our knowledge of nature, as Kant has done.*

This discussion of Kant on material idealism is not free from obscurity, on account of his leaving the reader to discover how far he is speaking of external phenomenal realities, in distinction from Berkeley's idealism, and how far of "*noumena*" or things in themselves. His succeeding remarks entitled, "*The Ground of the Distinction of all Objects into Phenomena and Noumena,*" may throw further light both on the realistic and the idealistic side of his system. The understanding, says our philosopher, can never make any use of its *à priori* conceptions and principles but what relates immediately to our sensuous experience of phenomena: it cannot make a transcendental use of them—that is, it cannot apply the categories and "principles" to things considered in themselves. Why have mathematical conceptions significance? Take the example: "between two points there can be only one straight line." This principle and its representation are generated in the mind wholly *à priori*; but it would mean nothing if we were not able to exhibit it empirically to sense. An abstract conception must be made sensuous—that is, an object must correspond with it in intuition, else the conception is void of meaning: and so throughout the entire domain of the understanding. Quantity is only explained by means of some adopted unit of sense, and we everywhere find such empirical illustrations necessary. In a word, no conception will have a corresponding object, if we take away sensuous intuition—the only intuition we possess.† Thought itself is only the act of referring a given intuition to an object; and, beyond the sphere of passible experience, no category and no synthetic *à priori* principle can apply.

Now as nothing but phenomena are presented to us in sense; that is, nothing but things as they appear to us—to these *phenomena,* alone, can the understanding apply the categories and the principles derived from them. Other possible things, which are not objects of our senses but are merely cogitated by the understanding, are *noumena* or things thought but not experienced. Kant distinguishes these abstractions or hypotheses which the understanding forms to itself, with nothing in the sensuous

* Sir W. Hamilton uses the term "consciousness" in a similar manner. The question simply relates to the nature of our convictions: is the certainty to me of my own thought and existence, of the *same kind* precisely as the certainty to me of the existence of objects around me—even granting that I am equally sure of both?

† This principle is the key to a large part of Kant's speculations on "Reason."

faculty corresponding with them, into *negative* and *positive*. The notion the understanding represents to itself of a substratum to certain sensible properties which sustains or holds them, as it were, together (itself being no object of sense), Kant calls a *negative* noumenon. It is an object of sense, considered in so far as it is not an object of our sensuous intuition. It is the supposed substance or base to which all the sensuous properties belong, regarded abstractedly. On the other hand, a *positive* noumenon is something which is not at all an object of sensuous intuition, but which the understanding is supposed to have the power of presenting to itself as an object of knowledge by means of a kind of intellectual intuition—a faculty which Kant wholly denies to man. Several serious consequences flow from his theory of "positive noumena," but they belong chiefly to his doctrine of "reason" as distinct from understanding. A stone considered in its *substance* merely, apart from its qualities, is a negative noumenon; an angel is a positive noumenon. He admits that the conception of these noumena, either positive or negative, is not self-contradictory; but he affirms that their posibility is, to us, incomprehensible, since all that is not phenomenon is to us a mere void. Understanding and sense must be conjoined; if we separate them, we suppose representations which we cannot apply to any determinate object.

It is evident that Kant's theory of positive noumena entirely limits our knowledge, properly so called, to what is sensuous, and borders on scepticism; he only avoids this rock by an inconsistent distinction between understanding and "practical reason," on which we cannot now enter. His views on noumena, negatively considered, are essentially connected with the entire subjectivity of his theory of perception. Not only are all secondary qualities—sound, heat, colour, taste, and the like, *in us* the subject of them; the same is the case with all attributes connected with space and time, such as magnitude, succession, length of duration, etc. These do not exist in things, they depend on our sensuous faculty. We give to all our perceptions a certain space and time, and we speak of objective validity. But when we abstract all time-and-space relations, there remains nothing that we can regard as belonging to the object in and by itself, excepting that it is some unknown $x$. Neither the categories of understanding, nor the intuitive perceptions of sense, either separately or combined, can be referred to anything objective but phenomena. We can know nothing of a noumenal or "intelligible" world. His theory of negative noumena borders narrowly on the "material idealism" which he is so anxious to distinguish from his own "critical" or "transcendental" idealism. Surely it was an assumption to maintain that things, as they are in themselves, are so different from what they are as manifested to us. If they are not as they appear to be—how do we know it? Very true we can only become acquainted with them by our senses; but their being sensuous proves them material in themselves. Their properties are their very nature; their phenomena are essential to them, although we may not detect all that is in them. It is a *petitio principii* to suppose that things, so far as known to us, are not revealed *as they are*, but in some other way. If our understanding and our senses are not conformed to the truth of things, they are of little avail to guide us to any kind of knowledge. While Kant limits our understanding entirely to sensuous things, he denies that even here we can have anything more than a relative and subjective knowledge. He denies that objective realities can be known, either by sense or understanding, or both combined, while yet he admits them, and makes their admission an important distinction of his system. He says he never doubted the existence of things, in themselves, (*Ding an sich,*) as distinct from mere phenomena: yet his whole theory of time and space as applied to what we regard as the objects around us, by rendering matter or substance (whatever be our speculations with regard to its nature as atomic or merely dynamical) wholly dependent on our subjective capacities, is barely distinguished from the idealism of Berkeley.

Our author follows up his transcendental distinction of phenomena and noumena by a dissertation on the equivocal character or *Amphiboly of the Conceptions of Reflection,** which arises from not observing that distinction, and thus confounding the objects of the pure understanding with those of sense. Reflection in general, with Kant, is that state of mind which inquires into the relation of our different representations, in regard to the faculty to which they subjectively belong—the understanding or the senses. Transcendental reflection is the act of determining whether our representations are compared with each other as originating in the pure understanding or in sensuous intuition. Kant terms the source of our conceptions, either in the one or in the other, the transcendental "*place*" of them. If we compare our conceptions together without inquiring to which place (faculty) their objects belong, whether as noumena to the understanding, or as phenomena to sense, we shall be liable to be deceived into error. Now the relations, says our philosopher, in which conceptions may stand towards each

* Chalybäus remarks that Kant's disciples have sometimes mixed up their own doctrines with their master's. Beck, one of his commentators, regards the *Reflexions-Begriffe* as categorical: Kant himself, however, says they are merely the comparison of representations as to their "place." They constitute, in fact, an immediate appendix to the remarks on phenomena and noumena.

other, in our minds, at the same time, are those of Identity and Difference, Agreement and Opposition, the Internal and the External, and the Determinable and the Determining (or Matter and Form). These four pairs of correlates he terms the Conceptions of Reflection (*Reflexions-Begriffe*); and what is to be avoided is an amphibolous (ambiguous) use of these relations —a confounding of the empirical use of the understanding as applied to things sensuous, with its transcendental use as applied to certain indeterminate objects of its own framing (noumena).

1. *Identity and Difference.* Suppose I think of two or more drops of water as mere conceptions occurring repeatedly to the understanding, abstracting the way in which these objects present themselves to the sensuous faculty; then the understanding aims in thought to seize these representations, and finds in fact no difference between them. The conception of a drop of water, as such simply, is always the same, however often repeated. But, to sense, this identity cannot present itself. As phenomena in space, the drops are not merely thought by the understanding, but each is intuited (perceived) by the sense as occupying its own space; and, abstracting all other considerations, this circumstance alone makes them numerically different. It follows that objects may be identical in regard to the bare conception of the understanding, while they are different in regard to sensuous intuition.

Hence, says Kant, the fallacy of Leibnitz's doctrine of the "*Identity of Indiscernibles;*" or that no two things, in nature, can be exactly alike; for, if they were, they would not be two, but one and the same. Now this is only true of our conceptions, not of objects as phenomena; for, grant it to be possible that any two things might in all other respects be alike, they must still differ because they must occupy different spaces. Leibnitz here confounded phenomena with things regarded as in themselves—objects of pure understanding, noumena.

2. *Agreement and Opposition.* In regard to these correlates, again, we may have confusion in our notions. Realities, as conceptions of the understanding (*realitas noumenon,*) says Kant, cannot be opposed. They cannot be so connected in one subject as to annihilate each other. Realities, as simple affirmations, are never in logical contradiction; in other words, no conception contains contrary affirmations. He means that, if you conceive of realities (things supposed as existing) logically only; you cannot unite in your conception of them opposite qualities excluding each other. The understanding may frame to itself the noumenal schema *triangle,* but not a triangle which has not three sides. On the contrary, the real in a phenomenon (*realitas phenomenon*)—real properties, may be opposed to each other while united in the same subject, so as wholly or in part to destroy each other. You might keep water on the fire, says Kant, at a given temperature, continually, by always adding to it ice: or two forces may act in the same straight line in opposite directions, and thus counteract each other: or a pleasure may balance a pain.

The Leibnitz-Wolfian philosophy maintained the principle, adopted by Leibnitz himself, that *Realities are never opposed to each other;* and this was the basis of his theory of evil in the Theodicæa. All evil, he said, is merely the absence of good (a reality). If good exists in the universe, evil cannot exist. It cannot be real, it must be a mere negation; for good and evil are opposed, and two realities cannot oppose each other. Hence Leibnitz regarded evil as simply a privation, the consequence of the limited nature of the creature; only in this way, said he, can evil find a place in the world along with good. Now, says Kant, it is true of our mere conceptions, in their logical character, that we cannot have contradictory elements; but it is not true of experience; it is not true of phenomena; and we know nothing of any things that are not phenomena: other things are mere hypotheses of the understanding. Real properties may be opposed, and are opposed in human experience. In this way Kant combats Leibnitz's argument that good is more real or positive than evil.

3. *The Internal and the External.* The interchange of these conceptions has also led to error. Our conception of substance or matter, as a phenomenon of external sense in space, is made up only of the properties of attraction and resistance. These determinations or forces acting, as we say, *in* it, are in truth merely external relations to other things; and the permanent phenomenon itself is merely a complex of these relations. But, on the other hand, when the understanding tries to conceive of substance in its essence, apart from all sensuous and external relations, as extension, impenetrability, contact, motion, and the like, (though this noumenon is to us really a *nescio quid,* or a nonentity,) the understanding must think of it, if at all, as having all its determinations absolutely internal. Its modifications have no relation to other things; they are only changes of our own internal state. Now, what other internal attribute of such an object as this can we think of, than that which our own internal sense presents to us—namely, the power of thought, or something having an analogy to it, such as the power of representation?

Our author brings to this test the *Monadology* and the *Pre-established Harmony* of Leibnitz; both which he regards as founded on an erroneous view of the difference between the internal and the external. Leibnitz makes the external relations of things as only apparent: their true designations are internal. The understanding grasps the elementary materials of the universe as simple abstractions, without parts, in short, *monads;* and finds that they can only have internal functions—namely, the power of "representing to themselves the universe," which they together constitute. And as the monads have thus no external relations, or any agency whatever on each other, the influences which we seem to see around us are not real. Objects, says Leibnitz, have no more influence among themselves than two clocks with no connecting mechanism, one of which points to the same hour which the other is striking. Hence the Pre-established Harmony, which accounted for the apparent effects of substances on each other, on the principle that the great First Cause, instead of interposing every instant and in every case, has fixed once for all the general laws by which substances are to correspond among themselves, though they have no real agency on each other.

4. *Matter and Form,* or the determinable and the determining. Our conceptions of these also require reflection, as to their relation to understanding or sense. *Matter* here signifies whatever may have determination given to it; *form,* its determination. In a conception of pure understanding something must be given, in order to its being determined. If I say A is B, the terms A and B are the given matter of the proposition, their relationship as expressed by the copula is the form. The matter must be known or thought before we can thus conjoin its elements by the mind. Hence, in the conception of the pure understanding, the matter or content must precede the form. But in the case of sense it is not so. Here, all objects are determined solely as phenomena; and the form of our intuitions, as a subjective quality of our sensuous faculty, must precede the matter or content of them: in other words, space and time are *à priori* in our sensuous nature, (for Kant, it will be remembered, makes them both sensuous,) and they precede all the phenomena of sense, that is, all the data of experience, and render them possible.

Now, according to Leibnitz, space is nothing but the order of things co-existing, and time the order of things successive. This is quite at variance with Kant's theory. Leibnitz erred, he says, by making space and time determinations of things themselves. Space was possible as a certain relation or order, in which substances have an apparent mutual communication; and time was the apparent sequence of their states, as causes and effects. He reasoned on the principle that the pure understanding can at once seize on objects and deduce their relations; whereas, it can only refer to objects by its categories, through the medium of sense, which in its forms of space and time is, *à priori,* the receptivity of all phenomena. Leibnitz* could not believe that the form could thus precede things, and determine their possibility; he therefore made space and time possible through our human view of the relationship of substances to each other. As to what is peculiar to space and time, and what seems independent of things, he ascribed this to an unavoidable confusion in our conceptions. He made space and time not only determinations of the monads (noumena); he also made them, as conceptions, valid of phenomena, because he sought everything in the understanding, not allowing to sense a peculiar mode of intuition, but assigning to it only the "despicable office" of confusing and disarranging the arrangements of the understanding.

The above remarks on the "Conceptions of Reflection," it will have been seen, are intended by our author to show the nullity of all conclusions obtained by comparing objects with each other in the understanding alone; for phenomena are the only things which can to our human faculties possess objective reality, and this because they give us intuitions to correspond with our conceptions. We can only know even ourselves, says Kant, through the intuition of the internal sense, consequently as phenomena; and we can discover nothing but phenomena in our existence, however we desire to penetrate into their non-sensuous cause. Leibnitz, in applying to things the principle that what belongs to or contradicts a conception in general, also belongs to or contradicts all that is particular which is contained under it, forgot that particular conceptions are such because they contain more than is thought in the general one. Thus the conception *eagle* contains more elements in its meaning than the more general conception *bird*. Yet, on the above principle, his whole intellectual system is built. In the mere general conception of a thing, he abstracted necessary conditions of sensuous intuition, and then treated these conditions as though they were not to be met with at all. Two drops of water, in this way, would be one and the same thing if they were always conceived of by the understanding as exactly alike; but this, as we have already seen, ignores their necessary numerical difference in space. Leibnitz thought he could know the internal nature of things, by comparing all objects in the understanding, and by means of abstract conceptions. He neglected sense, and regarded it as a confused mode of representation, always to be corrected by the understanding, and not as a primitive and special source of representa-

* The *Reflexionsbegriffe,* and their amphiboly, would almost seem to have been invented by Kant for the refutation of some of the main points in the speculative philosophy of Leibnitz.

tions. He was thus led to a pretended system of knowledge. True, says Kant, the understanding limits the sensuous faculty, but without enlarging its own sphere. Sense must not pretend to grasp things in themselves, but only phenomena. Understanding thinks to itself an object *in se*, but only as a transcendental one, which is the cause of the phenomenon—an abstraction which cannot be thought as quantity, reality, or substance, because these conceptions require always sensuous forms. This object is—we know not where—or whether it would disappear or not with the sensuous faculty. We call it a noumenon; but since we cannot apply to it any of the categories, its representation is for us empty and goes for nothing, excepting to mark out the limits of our sensuous faculty, and to leave remaining a void which we can neither fill by possible experience, nor by the pure understanding.

Kant regards as necessary to the completeness of his Transcendental Analytic, a brief statement of his views respecting the different ways in which we may arrive at the conception "*nothing*." He says we are capable of forming the conception of an object in general, problematically understood, without its being decided whether it is something or nothing. Now, in order to this decision, we must proceed according to the categories.

1. To those of *Quantity* (all, many, one,) *none* is opposed. This conception is *ens rationis*, an abstraction of reason, like *noumena*, which are not possible in the sphere of reality. This kind of nothing is *empty conception without object*.

2. In *Quality*, the real is something affirmed: negation is the opposite of reality; that is a conception of the absence of an object; cold, or a shadow, are examples. Here we have *nihil privativum*: this nothing is an object emptied of all reality, an *empty object of conception*.

3. Under *Relation*, we find the sub-category of condition or dependence. Pure space and pure time are no objects of intuition: they are mere forms or conditions on which phenomena depend, though this *ens imaginarium* is often spoken of as object. This kind of nothing is *empty intuition without object*.

4. In regard to *Modality*: the object of a conception which is self-contradictory is a mere non-entity, because the conception is impossible; as, for example, a figure constructed of only two straight lines. This sort of nothing is a *nihil negativum*, or an *empty object without conception*.

The reader may take these "nothings" as at least an example of Kant's ingenuity. They are cleverly made to correspond, in a certain sense, with the categories; so as to show the several ways in which we may arrive at the conception of *nothing*. Nor can the praise of great ingenuity and acuteness be denied to the developments which are contained throughout the "Analytic of Principles" according to the categories, whatever may be thought of some of the details. These details, however, are so mixed up with the categories themselves, that they to a considerable extent if not wholly depend on the merits of the latter.

This theory of the Categories has been severely criticized, both in Germany and elsewhere—not in regard to the idea, which is generally admitted, that thought must have its own constitution, and its ways of apprehending the objects of sense—but chiefly in regard to details. Indeed, although Kant's categories are merely subjective forms of the understanding, and applicable to phenomena alone, irrespectively of the question whether there be anything *real* as distinct from phenomena or not—nevertheless the categories are equally valid for the most dogmatic realism, so far as they are in themselves just. For whatever knowledge perception may give us, we must no doubt receive it according to the laws of the understanding as well as of the senses. The philosopher of Königsberg supposed that, like himself, the great Stagirite, in his ten categories, offered a subjective analysis of the elements of human understanding. Aristotle, however, aimed at the classification of external objects, though necessarily in relation to our faculties: Kant analyzed thought itself, in reference to objects. Kant's criticism of Aristotle's Categories (whatever be their own merits) is partly based on the error of supposing that his great predecessor's intention was exactly the same as his own; and he condemned Aristotle's list as a mere "rhapsody," and as hasty, incomplete, and confused. Kant took his categories from the arrangement of judgments, as he found it in the common logic; and he squared his list according to them. Hence he did not inquire into the relation of these abstract conceptions to each other as to their origin, but pronounced every one of them independent and irreducible. He said, for instance, that the conception of a *whole* was a combination of the conceptions of unity and plurality; while he nevertheless strenuously maintained that the third category in each set was as primitive and irreducible as the other two. In a similar way, he made *substance* and *cause* combine to produce "reciprocity of causation." Even if we admitted that the categories of the understanding actually present themselves to us under these Kantian forms, it should be asked: Have they always had these forms; if not, how did they assume them? In this way, it would surely be found that they are not all primitive and original, in the sense in which some of them are, but which he claims for all.

Independently of the principle on which our author proceeded, of adopting the ordinary logical forms of judgments as the basis of his pure conceptions of the understanding, without a special criticism in justification of this method, we may well hesitate to accept this list of categories as a whole: for, not to insist on other objections, it is evident that considerable difficulty attaches, in some cases, to our receiving each sub-category as essentially distinct from all the rest, though Kant himself attached vital importance to this point. For example, if we say "the soul is not mortal"—this is a "*negation;*" "the soul is non-mortal" is a "*limitation.*" One proposition denies that the soul is among mortal beings, the other affirms that it is among non-mortal beings; but then our negation has now become, in truth, an *affirmation* (reality): here then the categories of reality and limitation are confounded; and besides this, the meaning of the negation and the limitation is identical. A category of *predication,* in general, would, we venture to say, have absorbed all the three; and would obviously have been a much higher generalization. Again: *substance* and *existence* are given as pure conceptions wholly distinct from each other. Of course, we must take them both as only intended to refer to the phenomena of sense. Under the second "postulate of empirical thinking," Kant says: "Our knowledge of the *existence* of things reaches as far as our perceptions." Now may not exactly the same, according to his own theory, be said of *substances?* He expresses this postulate thus: "That which coheres with the material conditions of experience (sensation) is *real.*" Is there not, here, the absence of any definite distinction between existence and substance, and do they not mutually involve each other? Is there not, moreover, also a confounding of *reality* with substance? But reality, substance, and existence, are all arranged under different heads—Quality, Relation, and Modality. At all events, in several cases, the distinctions are not so sharply drawn as to prevent their being confounded; and it is difficult to avoid the conclusion that a considerable sacrifice has been made, unwittingly, to the seductive influence of method and symmetry. Our space does not admit of any further remarks: we will however add, that whatever imperfection may attach to this famous list of categories—which are the work of great, but perhaps (as Sir William Hamilton remarks) of "perverted ingenuity," it must be admitted this does not essentially affect his main point—that we can *know* objects only as they are related to our faculties of sense and understanding, and that all our knowledge is *subjective.* His conclusion from this principle, that *we cannot know anything of the supersensible* is especially connected with another part of his system, the Transcendental Dialectic.

